# Determinants of patient and physician global assessments of disease activity in anti-neutrophil cytoplasmic antibody-associated vasculitis

**DOI:** 10.3389/fmed.2023.1107148

**Published:** 2023-02-09

**Authors:** Marius Rohde, Anna Kernder, Hasan Acar, Christina Düsing, Rebecca Fischer-Betz, Isabell Haase, Johanna Mucke, Oliver Sander, Jutta Richter, Tim Filla, Matthias Schneider, Gamal Chehab

**Affiliations:** Medical Faculty, Department Rheumatology & Hiller-Research Unit Rheumatology, Heinrich-Heine-University Düsseldorf, Düsseldorf, Germany

**Keywords:** patient-reported outcome, disease activity, ANCA associated vasculitis, pain, global disease assessment

## Abstract

**Objective:**

To compare physician and patient assessments of global disease activity in anti-neutrophil cytoplasmic antibody (ANCA)-associated vasculitis (AAV) and to identify associated factors.

**Methods:**

Global disease activity scores (0–10 points) were retrospectively analyzed from physicians and patients with AAV at each outpatient visit from 2010 to 2020. We compared the scores and performed a linear regression with a random effects to identify associated factors.

**Results:**

Patients (*n* = 143, 1,291 pairs, 52% female) had a mean 64 (±15) years of age and a mean disease duration of 9 (±7) years. Patients and physicians global disease activity assessments showed a moderate correlation (Pearson R 0.31, CI [0.23–0.52], *p* < 0.001). Linear regression showed a strong association between the physician-documented disease activity scores and serum CRP levels (β = 0.22, CI [0.18, 0.28]), disease duration (β = −0.022, CI [−0.04,-0.01]) and patients’ assessment of disease activity (β = 0.08, CI [0.04, 0.12]). By contrast, patient assessments were strongly associated with the degree of pain (β = 0.30, CI [0.25, 0.35]), functional limitations in daily living (HAQ, β = 0.49, CI [0.21, 0.78]) and the global physical well-being (NRS, β = 0.39, CI [0.32, 0.46]).

**Conclusion:**

Patients’ and physicians’ assessments of disease activity correlated. High CRP levels and disease duration were associated with physician-assessed disease activity scores, while subjective limitations were associated with higher patient-assessed disease activity scores. These findings highlight and support the need to develop and evaluate patient-reported outcomes to assess disease activity in patients diagnosed with AAV.

## Introduction

Patient-reported outcomes (PROs) have become increasingly important in the assessment of rheumatic diseases in recent years. In systemic lupus erythematosus, they provide additional information on aspects of the disease that are not adequately addressed in clinical routine. In addition, patient-reported questionnaires empower the patients, can be a way to prioritize follow-up appointments, and allow the collection of a large amount of data with significant time and cost savings (1–4).

In ANCA-associated vasculitis (AAV), the generic Short-Form 36 (SF-36) measuring Health related quality of Life (HRQoL) was the only PRO included in the Outcome Measures in Rheumatology (OMERACT) core set for a long time (5–7). Due to the lack of specificity of generic PROs (8), the OMERACT Vasculitis Working Group identified the need for an AAV-specific PRO (9).

In 2018, the OMERACT Vasculitis Working Group developed and validated the ANCA-associated vasculitis and patient reported outcome (AAV-PRO) questionnaire.

While the AAV-PRO assesses disease-related limitations from the patient’s perspective, an investigation of whether the different domains of the questionnaire represent disease activity has not yet been performed. However, the assessment of disease activity is of particular importance for determining the need for follow up appointments, clinical/laboratory testing, and therapeutic decisions.

The aim of our study was to investigate patient’s assessment of their disease activity on a numeric rating scale (0–10), compare it with the physician’s assessment, and identify potential influencing factors, to support the development of PRO measures for disease activity in AAV and to contribute to the optimization of disease management.

## Methods

We performed a retrospective analysis of data from patients diagnosed with AAV [including those with granulomatosis with polyangiitis (GPA), eosinophilic granulomatosis with polyangiitis (EPGA), and microscopic polyangiitis (MPA)] who underwent treatment by board certified rheumatologists in our rheumatology outpatient clinic between 01 January 2010 and 31 December 2020. The data were routinely collected in our outpatient’s clinic at each visit in the given period. Patient and physician-rated global assessment of disease activity (PGA) were determined separately and independently at each patient visit, using a numeric rating scale (NRS; 1–10 points). We additionally regarded clinical factors that might influence this assessment, including patient age, sex, body mass index (BMI), disease duration, laboratory values, degree of pain (NRS 0–10), results of the Health Assessment Questionnaire (HAQ), and the physical and psychological well-being (NRS 0–10) of each visit. The Birmingham Vasculitis Activity Score [BVAS (V3.0)] measuring disease activity in vasculitis and the organ involvement were not routinely collected in our cohort. We had information about the BVAS in 20.9% of our patients and about the organ involvement in 48% of our patients. Remission status was indicated by the evaluation of the physician.

We performed a linear regression to analyze the association of the patient and the physician PGA scores of disease activity to one another as well as each of the aforementioned variables. Hereby, we included the patients-and the physician-ID as random effects to account for repeated enrolments of the same patients and the assessments by different physicians. The analyses was conducted with a random interceptor to account for patients/physicians individual degree of increase or decrease of disease activity. We additionally performed a sensitivity analysis using generalized additive linear regression (GAM analysis) to account for nonlinear relationships between the covariates and the PGA of disease activity.

Data were analyzed with the statistical software program R (The R Foundation for statistical computing, Vienna, Austria, Version 4.1.2). We visually checked for normal distribution. For normally distributed parameters, we used the mean and standard derivation [mean (±SD)], otherwise the median and interquartile range [median (IQR)] were reported.

This study was reviewed and approved by the Heinrich-Heine-University Duesseldorf Institutional Review Board (2021–1365). The study complies with the Declaration of Helsinki and required no additional review or approval.

## Results

### Study cohort

Our analysis included data from 143 patients who had been diagnosed with AAV. Most of the patients enrolled in our study were diagnosed with GPA (*n* = 107, 74.8%). Women represented 52.4% of the patient population. The mean age of the patient cohort was 64.0 years (±14.6), the mean BMI was 27.3 kg/m^2^ (±5.7), and the mean disease duration was 9.2 years (±6.6). The average score for pain experienced in the previous 7 days (NRS 0–10) was 2.5 points (±6.0). Additional details are included in Table 1.

**Table 1:** Characteristics of the patient cohort.

	*n* (%)	Mean (SD)	Median [IQR]
Individuals	143 (100)		
Visits	1,291 (100)		
Females	75 (52.4)		
GPA	107 (74.8)		
EGPA	22 (15.4)		
MPA	14 (9.8)		
Visits per patient		9.0 (6.7)	5 [6]
Age (years)		64.3 (14.6)	66 [21]
BMI		27.3 (5.7)	25.7 [6.6]
Patient-assessed global disease activity score (A)		2.6 (2.4)	2 [4]
Physician-assessed global disease activity score (B)		1.8 (1.1)	2 [2]
Difference (A-B)		0.8 (2.3)	0 [3]
Disease duration (years)		9.2 (6.6)	8 [9]
Pain past 7 days (NRS)		2.5 (6.1)	2 [4]
Global health status (NRS)		3.8 (2.1)	6 [3]
Psychologic well-being (NRS)		2.6 (2.1)	2 [3]
Functional status (HAQ Score)		1.0 (0.7)	0.8 [0.8]
CRP (mg/dl)		0.7 (1.1)	0.3 [0.5]

Global disease activity was assessed by patients **(A)** and physicians **(B)** during 1,291 clinic visits over a 10-year period. SD, standard deviation; IQR, interquartile range; GPA, granulomatosis with polyangiitis; EGPA, eosinophilic granulomatosis with polyangiitis; and MPA, microscopic polyangiitis.

One-hundred and forty-three patients and their treating physician assessed AAV disease activity at each outpatient visit; this resulted in 1,291 paired assessments. On average, each patient was seen and evaluated at 9.0 outpatient visits (±6.7). Most patients were constantly attended by one physician during their multiple outpatient visits [median change of the attending physician: 1 (IQR 1)].

### Comparison of patients and physicians global assessment of disease activity

The physician’s global assessment of disease activity correlated with BVAS Score (V3.0; R 0.49, p 0.0058), a validated questionnaire to assess disease activity by physician, whereas the patients global assessment of disease activity did not (*R* =  − 0.1, *p* = 0.6).

**Figure 1:**
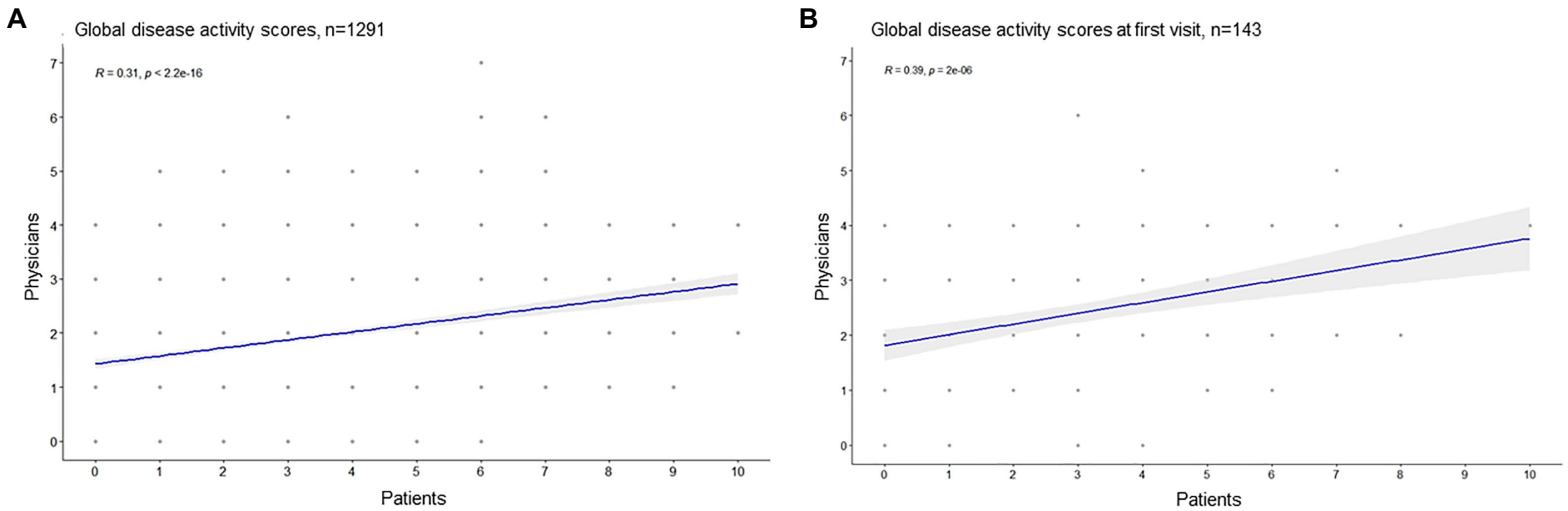
Correlation of patients and physicians global disease activity assessment (Pearson correlation). **(A)** All visits, **(B)** only the first documented visit.

**Figure 2:**
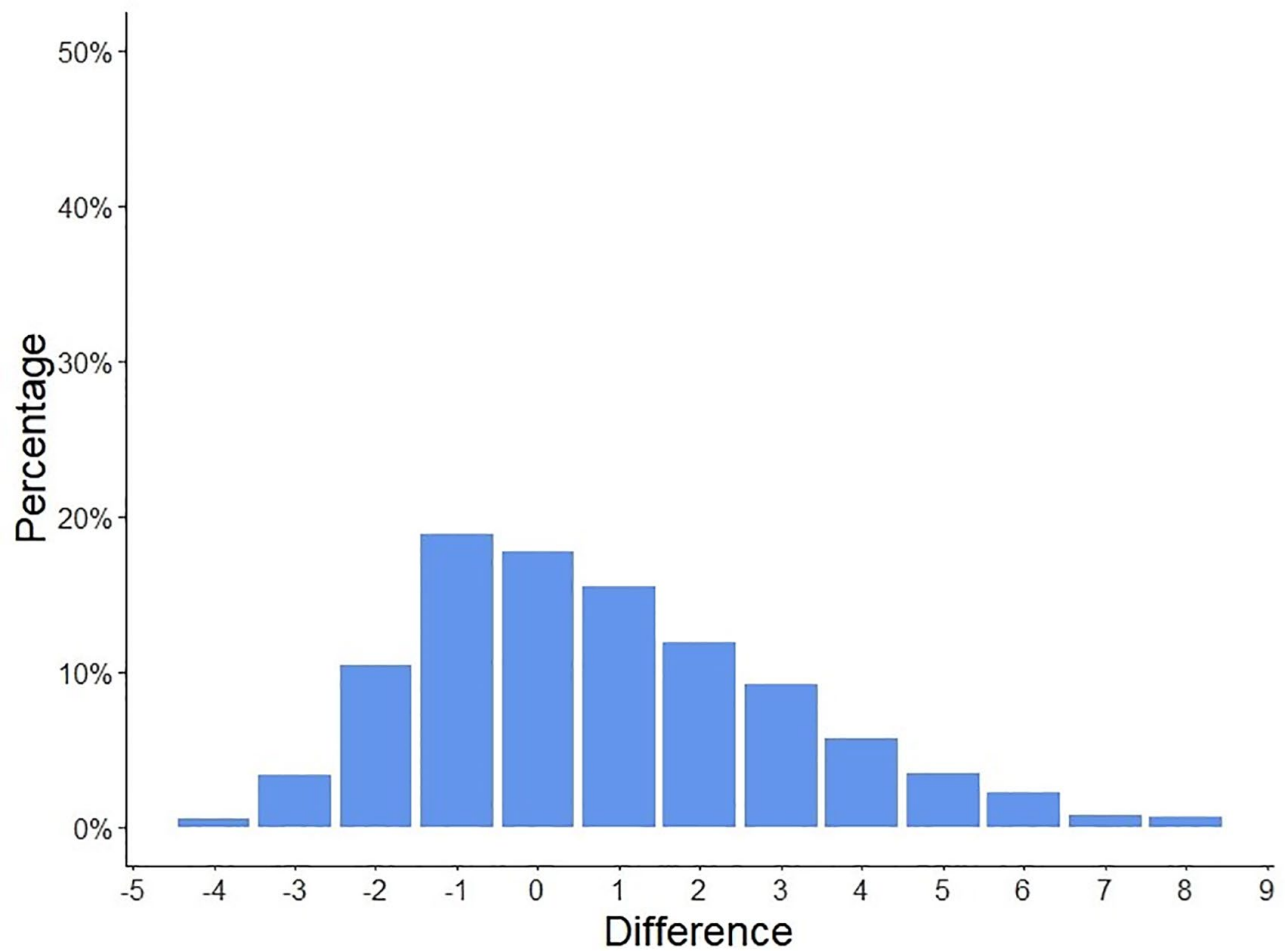
Differences in patient-assessed and physician-assessed global disease activity scores. The patient and physician-assessed scores diverged by ≤2 points in 66.3% of the cases (*n* = 856).

Patients and physicians global disease activity assessments showed a moderate correlation (Pearson R 0.31, CI [0.23–0.52], *p* < 0.001), the median PGA of disease activity assessed by both, patients and their physicians were two points, Figure 1. In 66.3% of the cases (*n* = 856), the patient and physician assessments diverged by ≤2 points, Figure 2.

**Figure 3:**
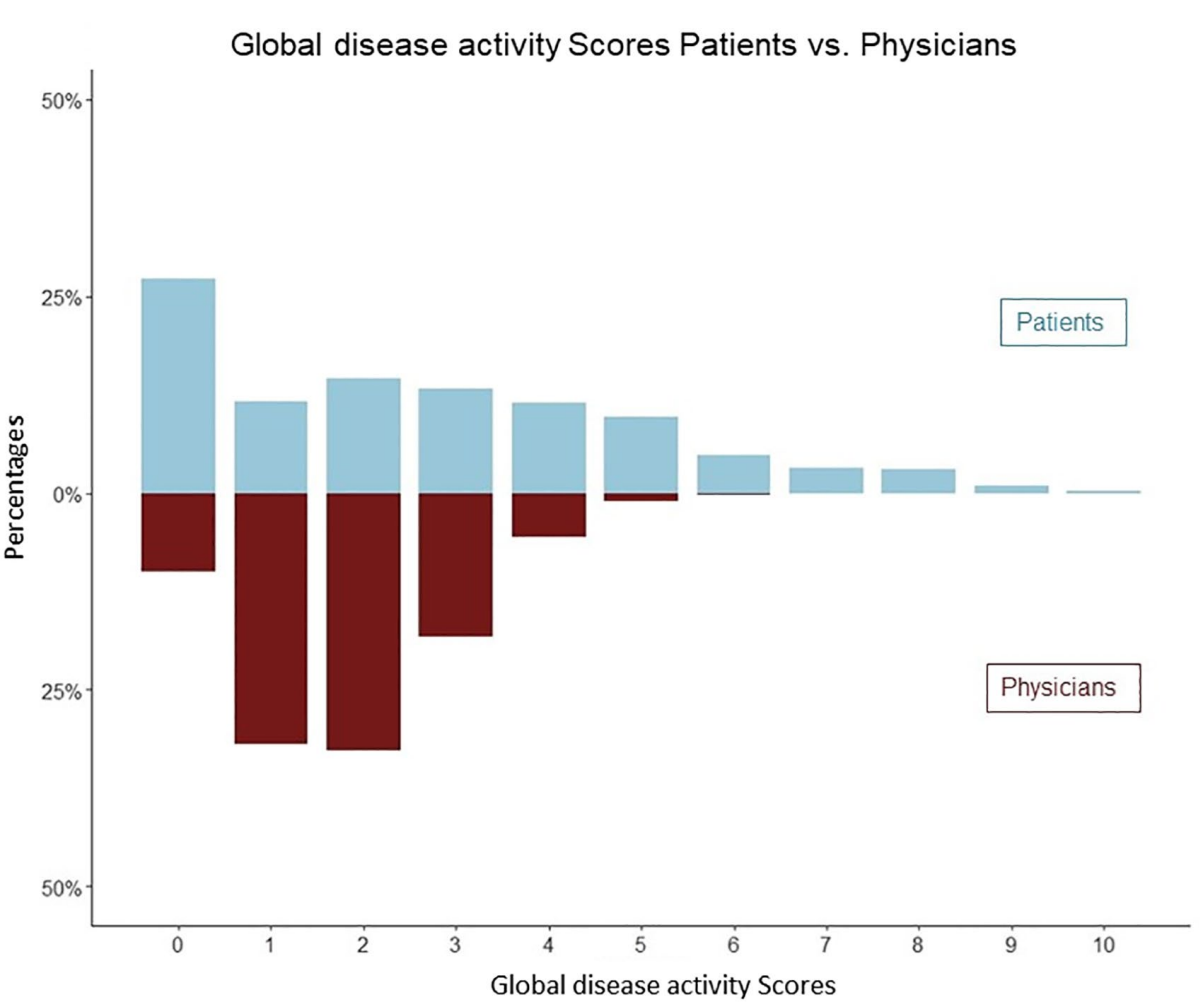
Distribution of physicians and patients global assessments of disease activity (mirror plot). Patients were more likely to assess a disease activity score of 0 points (*n* = 352) and greater than 5 points (*n* = 280).

Patients were more likely to assess a disease activity score of 0 points (*n* = 352) and of greater than 5 points (*n* = 280), compared to physicians (0 points *n* = 130, > 5 points *n* = 17), Figure 3. High levels of patients disease activity scores (5–10) did not correlate with the paired physicians assessments (Pearson R 0.03, CI [−0.09, 0.14], p 0.67).

The subgroup of patients that assessed their disease activity higher than their treating physicians were older (68.9 vs. 66.4 years, CI [2.72–6.35], *p* < 0.001), experienced more pain (NRS 3.9 vs. 1.1 CI [2.49–3.05], *p* < 0.001), reported more limitations in daily living (HAQ 1.2 vs. 0.7, CI [0.41–0.56], *p* < 0.001) as well as worse physical and psychological well-being (psychological well-being NRS 3.3 vs. 1.8, CI [1.26–1.75], *p* < 0.001), physical well-being (NRS 4.6 vs. 2.9, CI [1.46–1.92], *p* < 0,001) as compared to the subgroup of patients that assessed their disease activity lower than their physicians.

Patients who reported PGA of disease activity >5 points were older (69.2 vs. 65.7 years, CI [−5.3, −1.6], *p* = 0.03), reported having more pain (NRS 3.4 vs. 2.1, CI [−3.8, −3.1], *p* = 0.02), reported more limitations in daily living (HAQ 1.1 vs. 0.8, CI [−0.7, −0.5], *p* = 0.002), and reported higher levels of psychological distress (NRS 3.0 vs. 1.9 CI [−2.2, −1.6], *p* = 0.02) compared to patients who reported a PGA of disease activity of ≤5 points. BMI, serum CRP levels and disease duration did not differ between these groups.

### Global disease activity assessment in current flares

Patient and physician assessments of global disease activity differed depending on whether the patient was experiencing a flare or being in remission (patients CI [0.74, 2.22], *p* < 0.001; physicians CI [1.77, 2.66], *p* < 0.001). Both—patients and physicians—assessed disease activity at a median of four points during flares and two points while in remission. There was a pronounced correlation of patients and physicians disease activity scores in patients with ex-flares compared to patients who never experienced a flare [patients with ex-flares (*R* = 0.61 CI [0.31–0.79]) vs. who never experienced a flare (*R* = 0.30, CI [0.24–0.35])].

In 417 visits patients received Prednisolone [median dosage median 4 mg (IQR 2.5)]. We observed higher patients and physicians disease activity scores in patients taking glucocorticoids (independed of the dosage) compared to patients not taking prednisolone (Mean Patient GA 2.7 vs. 2.0, CI [0.37, 1.05], *p* < 0.001; Mean Physician GA 1.9 vs. 1.4 CI [0.29, 0.63], *p* < 0.001). The prednisolone dosage did not correlate with the patients or physicians global disease activity scores (patients *R* = 0.042, physicians *R* = 0.15), Pearson correlation. Next, we assessed the impact of the immunosuppressive therapy on the assessment of disease activity. Physicians assessed disease activity higher in patients taking Rituximab (2.1 vs. 1.8, CI [−0.46; −0.07], *p* = 0.007), whereas the patients and physicians disease activity scores were lower in patients taking Mycophenolate mofetil (patients scores: 2.0 vs. 2.6, CI [0.14; 1.11], *p* = 0.01; physicians scores: 1.4 vs. 1.8, CI [0.19; 0.69], *p* < 0.001).

Physicians scored the global disease activity higher in PR3-ANCA positive patients (1.9 vs. 1.2 CI [0.39; 1.02], *p* < 0.001), whereas the patients global disease activity assessment did not differ between PR3-ANCA/MPO-ANCA patients (2.2 vs. 2.9, CI [−1.63; 0.11], *p* = 0.8).

Regarding organ involvement, patients disease activity assessment was higher in patients with pulmonal involvement (1.8 vs. 1.0, CI [0.29, 0.63], *p* = 0.043) and lower in patients with ear nose throat (ENT) involvement (1.1 vs. 1.8, CI [−0.13, −0.27], *p* = 0.008). There were no differences in patient’s global disease activity assessment comparing the different organ manifestations (renal involvement, lung involvement, ENT involvement, and nervous system involvement).

### Determinants of global disease activity assessment

We performed a linear regression analysis with patients- and physicians-ID as random effects to examine the impact of several factors on both, the patient and physician assessments of global disease activity.

Among our findings, physician-documented disease activity was strongly associated with patients’ disease activity assessments (β = 0.08, CI [0.04, 0.12], *p* < 0.001), serum CRP levels (β = 0.22, CI [0.16, 0.28], *p* < 0.001), disease duration (β = −0.02, CI [−0.04, −0.01], *p* = 0.001), and slightly with patients’ physical well-being (β = 0.01, CI [0.05, 0.14], *p* < 0.001) and the BMI (β = 0.02, CI [0.00, 0.04], *p* = 0.014).

By contrast, patient disease activity assessments were strongly associated with the degree of pain (β = 0.30, CI [0.25, 0.35], *p* < 0.001), the functional limitations in daily living (HAQ, β = 0.49, CI [0.21, 0.78], *p* = 0.001), and the global physical well-being (NRS, *β* = 0.39, CI [0.32, 0.46], *p* < 0.001).

In addition, patients’ scores were inversely associated with BMI (β = −0.03, CI [−0.06, −0.00], *p* = 0.035), albeit only slightly, details are given in Table 2. A sensitivity analysis (GAM analysis) to account for nonlinear relationships between the covariates and the PGA of disease activity reported robust results. Relationship of variables are shown in Supplementary Figures 1, 2.

**Table 2:** Linear regression with random effects of patient and physician assessments of global disease activity.

Disease activity patient				Disease activity physician		
	Estimates	CI (95%)	*p*	Estimates	CI (95%)	*p*
Age	−0.00	−0.01 – 0.01	0.672	−0.00	−0.01 – 0.01	0.763
BMI	−0.03	−0.06 – −0.00	0.035	0.02	0.00–0.04	0.014
Sex [female]	−0.26	−0.60 – 0.08	0.129	0.12	−0.08 – 0.31	0.242
Disease activity physician (NRS 0–10)	0.22	0.11–0.33	<0.001			
Disease activity patient (NRS 0–10)				0.08	0.04–0.12	<0.001
Functional status (HAQ)	0.49	0.21–0.78	0.001	−0.22	−0.39 – −0.06	0.007
Pat phys. Well-being (NRS 0–10)	0.39	0.32–0.46	<0.001	0.10	0.05–0.14	<0.001
Pat. psych. Well-being (NRS 0–10)	0.05	−0.01 – 0.11	0.130	0.01	−0.03 – 0.05	0.610
Pain last 7 days (NRS 0–10)	0.30	0.25–0.35	<0.001	0.01	−0.03 – 0.04	0.758
Diagnose duration (years)	−0.01	−0.04 – 0.01	0.231	−0.02	−0.04 – −0.01	0.001
CRP (mg/dl)	0.08	−0.03 – 0.18	0.158	0.22	0.16–0.28	<0.001

BMI, body mass index; CRP, C-reactive protein; HAQ, health assessment questionnaire; NRS, numeric rating scale; and CI (95%) 95% confidence interval.

## Discussion

### Patient reported outcomes in AAV

The AAV-PRO was recently evaluated and validated. It assesses the disease-related limitations from the patient’s perspective (9) but an investigation of whether the different domains of the questionnaire represent disease activity has not yet been performed.

For physicians, a validated tool for the assessment of disease activity in vasculitis exists [Birmingham Vasculitis Activity Score (BVAS)]. It is known that the BVAS Score correlates with the physician’s global disease activity on a numeric rating scale (10). In our cohort, we were able to confirm this correlation.

Our study aimed to examine and compare the results of physician and patient global assessments of disease activity and to identify associated factors. Our findings are of interest to support the development of PRO measures for disease activity in AAV and to contribute to the optimization of disease management.

### Comparison of patients and physicians global assessment of disease activity

Our findings revealed that most of the patients (66.3%) and their physicians assessed disease activity similarly, yielding a median difference of ≤2 points. But, as already reported for other rheumatic diseases (11, 12), patients were more likely to assess a higher disease activity.

The correlation between patient and physician assessments was stronger in situations in which disease activity was comparatively low. These results are comparable to those reported by Harris et al. (3) who found that patient and physician assessments in Systemic lupus erythematosus (SLE) correlated with one another at low but not at high levels of disease activity. Likewise, this effect was shown for Rheumatoid Arthritis (12, 13), whereas an opposite trend was observed in the validation of the German Systemic Lupus Activity Questionnaire (SLAQ; for disease activity assessment in SLE (14)).

### Determinants of global disease activity assessments

As already shown for SLE (1, 2), we found that both patient and physician assessments of disease activity clearly differentiated between flares and remission, with median scores of four and two points, respectively. However, our study was conducted in our outpatient clinic and we did not enroll patients with highly active disease requiring hospitalization.

Our linear regression analysis revealed a strong association of the physician assessments of disease activity with serum CRP levels (as described before (15, 16)), this association was not observed in the patient assessments of disease activity. Compared to patients, who had no information about the laboratory results while assessing their disease activity, physicians had the possibility to check the laboratory results at the time of entering their disease activity assessment. As for each visit a new session is created in our clinical information system it is unlikely that the physicians and patients look back at previous disease activity assessments impacts the assessment of the disease activity.

In addition, the physician’s disease activity assessment was associated with the disease duration (lower disease activity assessments with longer disease duration). This association was previously reported for SLE which, like AAVs, is a chronic disease that can be relapsing (4).

By contrast, patient assessments were associated with the degree of pain, the global physical well-being and the functional limitations in daily living. Thus, subjective reports appear to influence the patient assessment of disease activity more than that provided by the physicians. This finding is also similar to that previously described for other rheumatic diseases (3, 17, 18). The importance of subjective reports was even more evident among patients who assessed their disease activity scores at >5 points.

We recommend to directly address the topics “*pain and physical well-being*” in the physician-patient conversation as our data revealed that they strongly affected the patient’s assessment of disease activity. Within this conversation, patients and physicians can evaluate whether the perceived pain and impaired physical well-being are an expression of increased disease activity. In addition, ways of reducing pain and improving physical well-being can be discussed (such as optimized analgetic therapy and physiotherapy) to improve the patient’s health related quality of life.

## Limitations

It is yet an unmet need to compare the global disease activity and BVAS with the domains outlined in the AAV-PRO. As our study featured a retrospective analysis of data collected over the past 10 years, we could not include these types of findings in our study.

In addition, the prediction of flares by the patient’s global disease activity assessment cannot be answered with our study cohort as in the majority of cases the last visit before a flare in our outpatient clinic was about 6 months before the flare. A prospective study, routinely assessing the patient’s global disease activity assessment f.i. every 4 weeks is warranted to address this important question. The majority of patients in our cohort received glucocorticoids in a dosage ≤5 mg/day. This dosage was not reduced depending on the patients or physicians global disease activity assessment in our cohort. However, as expected we observed higher patients and physicians disease activity scores in patients taking glucocorticoids compared to patients not taking them.

These important unmet needs of PROs are planned to be addressed by future prospective analyses including also data from other university and non-university-based clinics.

## Conclusion

In our study cohort, patients and physicians rated the disease activity equally, although patients were more likely to report higher levels (>5 points). Physicians’ and patients’ assessments of disease activity were associated with different factors, including CRP-levels and disease duration with the physician’s assessment and subjective limitations (pain, functional limitations in daily living and physical well-being) with the patient’s assessment. Consideration of all of these factors is important in the management of the disease. Our findings support the need to develop and evaluate patient-reported outcomes to assess disease activity in patients diagnosed with AAV.

## Data availability statement

The raw data supporting the conclusions of this article will be made available by the authors, without undue reservation.

## Ethics statement

The studies involving human participants were reviewed and approved by Ethics Committee of the medical faculty of the University of Duesseldorf, Germany (2021–1365). The patients/participants provided their written informed consent to participate in this study.

## Author contributions

MR, AK, HA, CD, RF-B, IH, JM, OS, JR, TF, MS, and GC designed the study. AK and MR performed the data analyses. AK and GC drafted the first version of the manuscript. All authors were involved in the critical interpretation of the results, discussed the findings together, critically reviewed the manuscript, and approved its final version.

## Conflict of interest

The authors declare that the research was conducted in the absence of any commercial or financial relationships that could be construed as a potential conflict of interest.

## Publisher’s note

All claims expressed in this article are solely those of the authors and do not necessarily represent those of their affiliated organizations, or those of the publisher, the editors and the reviewers. Any product that may be evaluated in this article, or claim that may be made by its manufacturer, is not guaranteed or endorsed by the publisher.
