# Supplementary figures and images for: Determinants of patient and physician global assessments of disease activity in anti-neutrophil cytoplasmic antibody-associated vasculitis
Source: Front Med (Lausanne). 2023 Feb 9;10:1107148. doi: 10.3389/fmed.2023.1107148 (PMC9947502; doi:10.3389/fmed.2023.1107148)

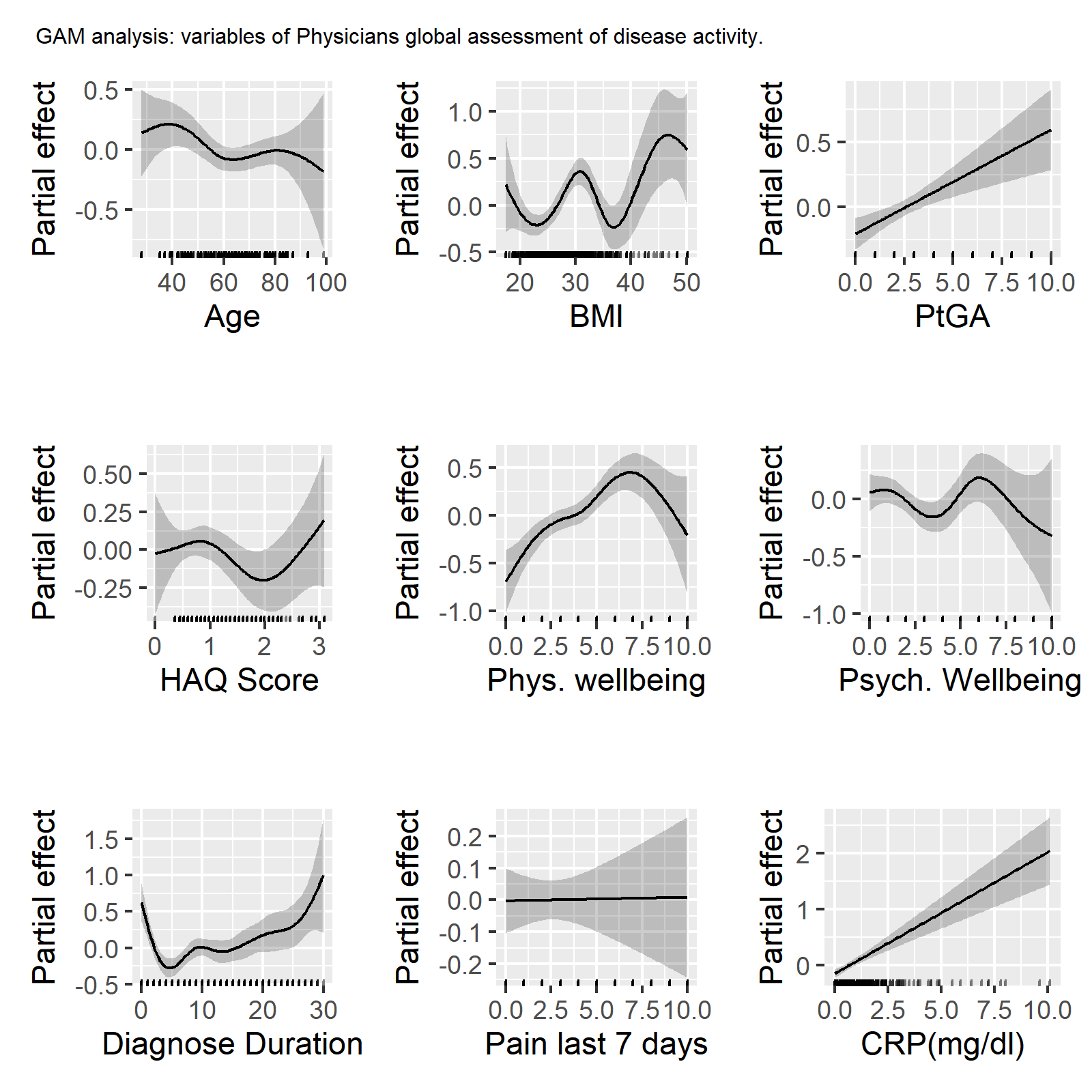

Supplement: Supplementary file 1 [file Image_1.tiff]

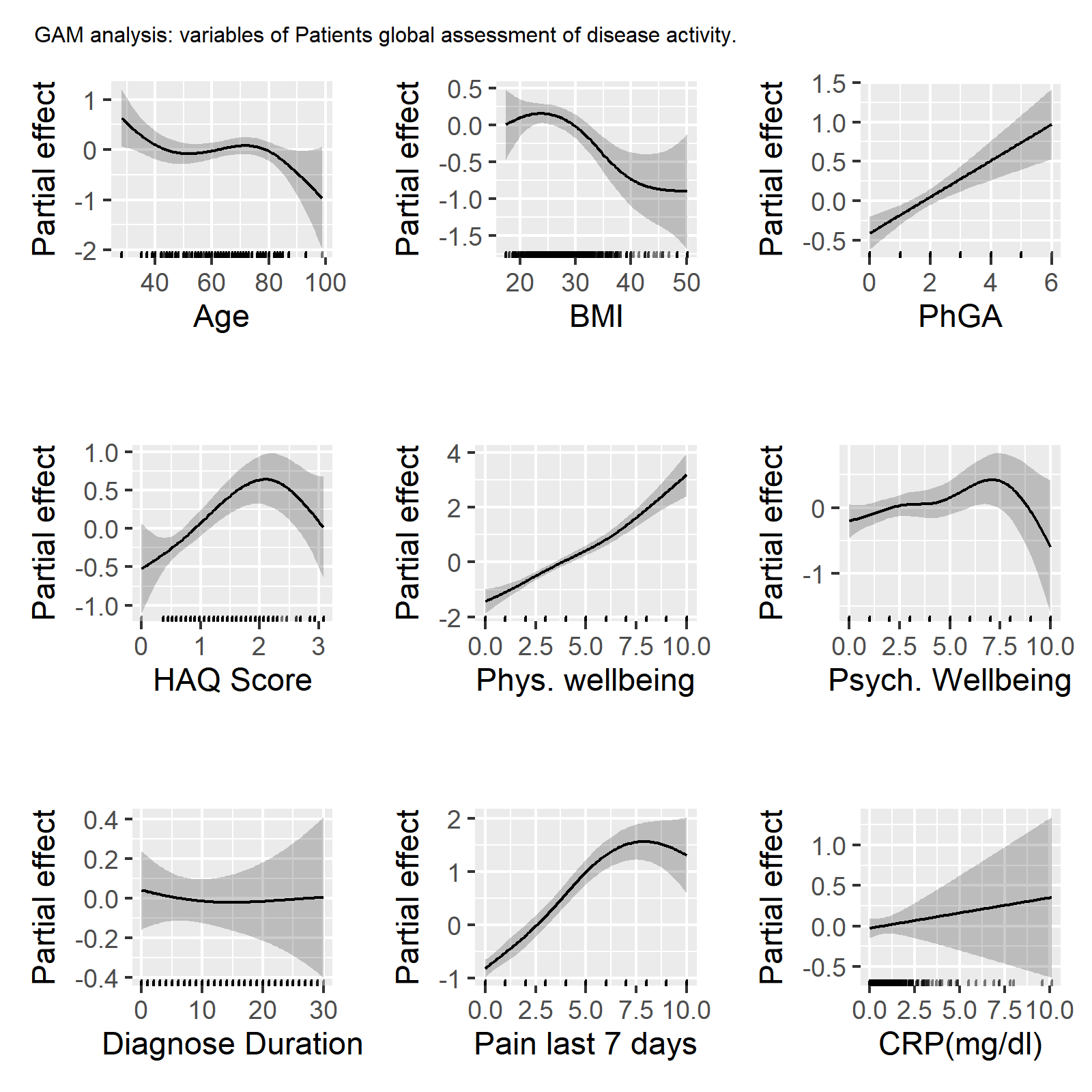

Supplement: Supplementary file 2 [file Image_2.tiff]
